# Matroidal Entropy Functions: A Quartet of Theories of Information, Matroid, Design, and Coding

**DOI:** 10.3390/e23030323

**Published:** 2021-03-09

**Authors:** Qi Chen, Minquan Cheng, Baoming Bai

**Affiliations:** 1 State Key Laboratory of Integrated Service Networks, Xidian University, Xi’ an 710071, China; 2 Guangxi Key Lab of Multi-Source Information Mining & Security, Guangxi Normal University, Guilin 541004, China

**Keywords:** entropy function, matroidal entropy function, matroid, orthogonal array, variable strength orthogonal array, almost affine code, MDS code, polymatroid

## Abstract

In this paper, we study the entropy functions on extreme rays of the polymatroidal region which contain a matroid, i.e., matroidal entropy functions. We introduce variable strength orthogonal arrays indexed by a connected matroid *M* and positive integer *v* which can be regarded as expanding the classic combinatorial structure orthogonal arrays. It is interesting that they are equivalent to the partition-representations of the matroid *M* with degree *v* and the (M,v) almost affine codes. Thus, a synergy among four fields, i.e., information theory, matroid theory, combinatorial design, and coding theory is developed, which may lead to potential applications in information problems such as network coding and secret-sharing. Leveraging the construction of variable strength orthogonal arrays, we characterize all matroidal entropy functions of order n≤5 with the exception of $\log10\cdot U_{2,5}$ and $\log v\cdot U_{3,5}$ for some *v*.

## 1. Introduction

Given N:={1,2,⋯,n} and discrete random vector $X:=(X_{i}:i\in N)$, the set function $h_{X}:2^{N}\to R$ defined by

$$h_{X}\left(A\right)=H\left(X_{A}\right),\;\forall A\subseteq N$$

is called the entropy function of X, where $X_{A}:=\left(X_{i}:i\in A\right)$ and $H(X_{\emptyset })=0$ by convention. We also say X characterizes $h_{X}$, or X is the characterizing random vector of $h_{X}$. An entropy function h can be considered as a vector in the entropy space $H_{n}:=R^{2^{N}}$. (For a set *A* and a finite set *B*, $A^{B}$ denotes the |B| Cartesian product of *A* with each coordinate indexed by b∈B. When A=F is a field, $F^{B}$ is a |B|-dimensional vector space over F with each coordinate indexed by b∈B.) We say $H_{n}$ and the vectors in it have order *n*. The set of all entropy functions of order *n*, denoted by $\Gamma_{n}^{*}$, is called the entropy region of order *n*. The closure of $\Gamma_{n}^{*}$, denoted by $\bar{\Gamma_{n}^{*}}$, is called almost entropic region. It is a convex cone [1]. A vector $h\in H_{n}$ is called entropic if $h\in \Gamma_{n}^{*}$, almost entropic if $h\in \bar{\Gamma_{n}^{*}}$, and non-entropic if $h\notin \Gamma_{n}^{*}$. Characterization of entropy functions, i.e., for a vector $h\in H_{n}$, determining whether it is in $\Gamma_{n}^{*}$ or $\bar{\Gamma_{n}^{*}}$, is of fundamental importance in information theory.

For a vector $h\in H_{n}$, if it is nonnegative, i.e., h(A)≥0 for all A⊆N, monotone, i.e., h(A)≤h(B) for all A⊆B⊆N, and submodular, i.e., h(A∩B)+h(A∪B)≤h(A)+h(B) for all A,B⊆N, the pair (N,h) is called a polymatroid, where *N* is the ground set and h is the rank function of the polymatroid. For a polymatroid (N,h), if h(A)∈Z and h(A)≤|A| for all A⊂N, (N,h) is called a matroid. By frequent abuse of terminology, we do not distinguish a (poly)matroid and its rank function if there is no ambiguity. See Section 2.1 for a more detailed discussion on matroids.

The set of all polymatroids in $H_{n}$, denoted by $\Gamma_{n}$, is called the polymatroidal region of order *n*. It is proved in [2] that any entropy function is a polymatroid, thus $\Gamma_{n}$ is an outer bound of $\Gamma_{n}^{*}$. As $\Gamma_{n}$ is closed, it is also an outer bound of $\bar{\Gamma_{n}^{*}}$. As inequalities bounding $\Gamma_{n}$ are equivalent to the nonnegativity of Shannon information measures, they are called Shannon-type information inequalities, and $\Gamma_{n}$ is also called the Shannon outer bound of $\Gamma_{n}^{*}$ and $\bar{\Gamma_{n}^{*}}$. For more about entropy functions and information inequalities, readers are referred to [3], (Chapter 13–15) [4,5].

It is well known that $\bar{\Gamma_{n}^{*}}⊊\Gamma_{n}$ when n≥4 due to the existence of non-Shannon-type inequalities, e.g., Zhang-Yeung inequality [6]. However, though $\bar{\Gamma_{3}^{*}}=\Gamma_{3}$, Zhang and Yeung also discovered that on an extreme ray of $\Gamma_{3}$, only countably many vectors are entropic, which implies that $\Gamma_{3}^{*}⊊\bar{\Gamma_{3}^{*}}$, and therefore there exists a gap between $\Gamma_{n}^{*}$ and $\bar{\Gamma_{n}^{*}}$ [1]. Given a random vector $X=(X_{1},X_{2},X_{3})$ with $X_{i},i=1,2,3$ mutually independent and each of them the function of the other two, it is proved in [1] that $X_{i}$ must be uniformly distributed on a finite set, say $Z_{v}:=\left\{0,1,\cdots ,v-1\right\}$, thus $h_{X}\left(A\right)=\log v\cdot \min\left\{2,|A|\right\},A\subseteq \left\{1,2,3\right\}$. On the other hand, for each integer v≥1, they proved that polymatroid h with h(A)=logv·min{2,|A|},A⊆{1,2,3} is entropic: let $X_{1}$ and $X_{2}$ uniformly distributed on $Z_{v}$ and $X_{3}\equiv X_{1}+X_{2}\;\left(\mathrm{mod}\;v\right)$, then h is the entropy function of $\left(X_{1},X_{2},X_{3}\right)$.

As the rank function of $U_{2,3}$ is equal to min{2,|A|},A⊆{1,2,3}, Zhang-Yeung indeed proved that for any vector $h=c\cdot U_{2,3}$ on the ray $R_{U_{2,3}}:=\left\{c\cdot U_{2,3}:c\geq 0\right\}$, h is entropic if and only if c=logv for some positive integer *v*. In [7], Matúš proved that, for any extreme ray $R_{M}:=\left\{c\cdot M:c\geq 0\right\}$ of $\Gamma_{n}$ containing a connected matroid *M* with rank ≥2, h=c·M is entropic only if c=logv for some positive integer *v*. However, on the other hand, h=c·M is not entropic for all positive integers. For example, we will see in Section 4 that $h=\log v\cdot U_{2,4}$ is non-entropic when v=2,6.

**Definition** **1.** *For a connected matroid M with rank ≥2, we call the set $\chi_{M}$ of all positive integers v such that h=logv·M is entropic the*probabilistically (p-)characteristic*set of M.*

The term p-characteristic set of a matroid *M* is first coined in [7]. As discussed above, $\chi_{U_{2,3}}=Z^{+}$, the set of all positive integers, and $\chi_{U_{2,4}}=\left\{v\in Z^{+}:v\neq 2,6\right\}$. In this paper, we study the p-characteristic set of an arbitrary connected matroid with rank ≥2.

**Definition** **2.** *For a connected matroid M with rank ≥2 and a positive integer v, if $v\in \chi_{M}$, we call the entropy function h=logv·M a* matroidal entropy function* induced by M with degree v.*

It can be seen in the proof of Zhang-Yeung, characterizing random vectors of matroidal entropy functions on $R_{U_{2,3}}$ is constructed by the multiplication table of an additive group on $Z_{v}$. It is not difficult to see that a random vector constructed by any quasigroup on $Z_{v}$, or equivalently, a Latin square with symbols in $Z_{v}$, or equivalently, an orthogonal array OA(2,3,v), characterizes $\log v\cdot U_{2,3}$. (See Section 2.2 for the definition of an orthogonal array.) More generally, an OA(t,n,v) can be used to construct a characterizing random vector of the matroidal entropy function $\log v\cdot U_{t,n}$ with t≥2. It is a natural question to ask whether such construction can be generalized to an arbitrary connected matroid *M* with rank ≥2? In [8], partition-representations $\xi_{i},i\in N$ of a matroid M=(N,r) with degree *v* was defined, where each $\xi_{i}$ is partition of a set Ω with cardinality $v^{h(N)}$. See more details in Section 3.1.2. Characterizing random vectors of h=logv·r can be obtained by the uniform distributions on the blocks of $\xi_{i}$. In [9], an equivalent definition in coding theory called almost affine code was defined. In this paper, in coordinate with the language in combinatorial design, we introduce variable strength orthogonal arrays(VOA) indexed by matroid *M* and integer v≥2, which is equivalent to a partition-representation of *M* with degree *v* and an (M,v) almost affine code. We denoted it by VOA(M,v). A VOA(M,v) can be regarded as expanding the concept of orthogonal array. If a VOA(M,v) exists, we will prove that the matroidal entropy function logv·M is entropic and a characterizing random vector of logv·M can be constructed by VOA(M,v). On the other hand, if VOA(M,v) does not exist, logv·M is non-entropic.

It is well known that orthogonal arrays with index unity in design theory are equivalent to maximum distance saperable (MDS) codes in coding theory. In discussions of our paper, we also see a more generalized equivalence, i.e., the equivalence between a VOA and an almost affine code. Thus, we review and develop the correspondences and equivalences in literatures such as [8,9] among four fields, i.e., information theory, matroid theory, combinatorial design, and coding theory, which may help them benefit from each other. In this paper, VOAs are also leveraged to characterize matroidal entropy functions induced by matroids of order n≤5.

The rest of this paper is organized as follows. Section 2 gives the preliminaries on matroid theory and orthogonal arrays. In Section 3, we first define variable strength orthogonal arrays and show their equivalence to the partition representation of a matroid and almost affine codes. Then we characterize matroid entropy functions via variable strength orthogonal arrays. in Section 4, we characterize matroidal entropy functions of order n≤5. A discussion of the applications and further research is in Section 5.

## 2. Preliminary

### 2.1. Matroids

There exist various cryptomorphic definitions of a matroid. In this paper we discuss matroid theory mainly from the perspective of rank functions. For a detailed treatment of matroid theory, readers are referred to [10,11]. In Section 1, we defined matroids as special cases of polymatroids. Here we restate the definition in the following.

**Definition** **3.** *A* matroid *M is an ordered pair* (N,r), *where the* ground set* N is a finite set and the* rank function r *is a set function on* $2^{N}$, *and they satisfy the conditions that: for any* A,B⊆N,

- 0≤r(A)≤|A| and r(A)∈Z,
- r(A)≤r(B),ifA⊆B,
- r(A)+r(B)≥r(A∪B)+r(A∩B).

*The value r(N) is called the* rank* of M.*

With a slight abuse of terminology and notations, we do not distinguish a matroid and its rank function. So $M,r_{M}$ and r may all denote the rank function of *M* when there is no ambiguity.

**Definition** **4.** *For integer n≥1 and 0≤t≤n, the* uniform matroid*$U_{t,n}$ with rank t and order n is defined by*

$$U_{t,n}\left(A\right):=\min\left\{t,|A|\right\}\;\forall A\subseteq N.$$

Given a matroid M=(N,r), for i∈N, if r(i)=0, element *i* is called a loop of *M*. For A⊆N, if r(A)=1, we call *A* a parallel class. If |A|≥2, the parallel class is called non-trivial. A matroid is called simple if it contains no loops and no non-trivial classes. For a matroid *M*, if we delete its loops and in each non-trivial parallel class, we delete all elements but one, then we obtain a simple matroid $M^{\prime }$. We call $M^{\prime }$ the simplification of *M*.

For a matroid M=(N,r), a nonempty C⊆N is called a circuit with size |C| of *M* if r(C−x)=r(C)=|C|−1 for any x∈C. It can be seen that any loop of *M* is a circuit of size 1 and a parallel pair {i,j} is a circuit of size 2. For a uniform matroid $U_{t,n}$, circuits are exactly those (t+1)-subsets *C* of *N*. In particular, $U_{0,n}$ contains *n* loops, any two elements of $U_{1,n}$ are parallel, and the ground set of $U_{n-1,n}$ forms the unique circuit of $U_{n-1,n}$.

**Definition** **5.** *A matroid is* connected *if any two elements in the ground set are contained in a circuit.*

It is easy to be verified that any uniform matroid $U_{t,n}$ with 1≤t≤n−1 is connected. This is because any x∈N is contained in a t+1 subset of *N* which is a circuit of $U_{t,n}$.

An extreme ray *R* of a convex cone *C* is a subset of *C* and for any r∈R such that $r=c_{1}+c_{2}$ and $c_{1},c_{2}\in C$, we have $c_{1},c_{2}\in R$, where $c_{1}=ar$ and $c_{2}=\left(1-a\right)r$ for some a∈R.

**Lemma** **1.** *[12] A loopless matroid is connected if and only if M is contained in an extreme ray of $\Gamma_{n}$.*

Each extreme ray of $\Gamma_{n}$ contains an integer-valued polymatroid, some of which are matroids. Such a matroid on an extreme ray is either a loopless connected matroid as stated in the above lemma, or a matroid obtained by adding loops to a connected matroids.

### 2.2. Orthogonal Arrays

Orthogonal array is a well studied topic in design theory. In this paper, orthogonal arrays are leveraged to characterize matroidal entropy functions. For a detailed treatment of this topic, readers are referred to [13].

**Definition** **6.** *A $\lambda v^{t}\times n$ array T with entries from $Z_{v}$ is called an* orthogonal array *of* strength *t,* factor *n,* level *v and* index *λ if for any $\lambda v^{t}\times t$ subarray $T^{\prime }$ of T, each t-tuple in $Z_{v}^{t}$ occurs in the rows of $T^{\prime }$ exactly λ times. We call T an $\mathrm{OA}(\lambda \times v^{t};t,n,v)$. When λ=1, we say such orthogonal array has* index unity *and call it an OA(t,n,v) for short.*

By the definition, for any $1\leq t^{\prime }<t$, an $\mathrm{OA}(\lambda \times v^{t};t,n,v)$ is also an $\mathrm{OA}(\lambda^{\prime }\times v^{t^{\prime }};t^{\prime },n,v)$, where $\lambda^{\prime }=\lambda v^{t-t^{\prime }}$. In this paper, we only consider the strength of the orthogonal array largest possible.

An important research problem of orthogonal arrays is the existence of an OA(t,n,v). The following lemmas state some results of this problem, in which Lemmas 2–4 can be found in Handbook [14].

**Lemma** **2** ([14], (III.7.16)). *There exists an OA(t,t+1,v) for any $v\in Z^{+}$.*

**Lemma** **3** ([14], (III.3.28, III.3.39)). *For $v\in Z^{+}$, an OA(2,4,v) exists if and only if v≠2,6.*

The nonexistence of OA(2,4,6) in Lemma 3 is the famous Euler’s 36 officer problem.

**Lemma** **4** ([14], (III.3.28, III.3.36, III.3.39)). *An OA(2,5,v) exists for all $v\in Z^{+}$ with three exceptions v=2,3,6 and one possible exception v=10.*

**Lemma** **5.** *For v=2,3,6, there does not exist an OA(3,5,v).*

This lemma is a folklore in the combinatorial design community. For self-contain, we prove it in the following.

**Proof.**  We prove the non-existence of OA(3,5,v) for v=2,6 by contradiction. Assume there exists an OA(3,5,2)A, i.e., a $2^{3}\times 5$ array whose each $2^{3}\times 3$ subarray contains each 3-tuple in $Z_{2}^{3}$ as a row exactly one time. By permuting the rows of A, we obtain an OA(3,5,2)$A^{\prime }$ such that the entries in the first $2^{2}$ rows and the 5-th column of $A^{\prime }$ are all 0. Let $c_{i},1\leq i\leq 5$ be the 5 columns of $A^{\prime }$ and $c_{i}^{\prime }$ be the vector consisting of the first $2^{2}$ entries in $c_{i}$. Now consider the subarray $\left[c_{i},c_{j},c_{5}\right]$ with 1≤i<j≤4. As its rows are exactly all 3-tuples in $Z_{2}^{3}$ and $c_{5}^{\prime }$ is a zero vetor, it can be seen that the rows of $\left[c_{i}^{\prime },c_{j}^{\prime }\right]$ are exactly all 2-tuples in $Z_{2}^{2}$. Thus, $\left[c_{1}^{\prime },c_{2}^{\prime },c_{3}^{\prime },c_{4}^{\prime }\right]$ forms an OA(2,4,2) which contradicts Lemma 3. The non-existence of OA(3,5,6) can be proved similarly.

For OA(3,5,3), assume such an array B exists. As each $3^{3}\times 3$ subarray of B contains each 3-tuple in $Z_{3}^{3}$ as a row exactly one time, for each two rows of the $3^{3}=27$ rows of B, their Hamming distance must be ≥3. Therefore, any two Hamming balls with center a row of B and radius 1 are disjoint. As there are 27 such Hamming balls with each size 11, there are at least 27×11=297 5-tuples, which contradicts the fact that only $3^{5}=243$ 5-tuples exist.  □

**Lemma** **6** ([15]). *If v≥4 and v≢2(mod4), then there is an OA(3,5,v).*

**Lemma** **7** ([16]). *Let x be an arbitrary odd positive integer. Let g be an arbitrary positive integer whose prime power factors are all ≥7 such that g≡3(mod4). Then*

*1.*  there is an OA(3,5,v) with v=35xg+5, if x≡1(mod4);
*2.*  there is an OA(3,5,v) with v=35xg+7, if x≡3(mod4).

## 3. Characterizing Matroidal Entropy Functions via Voa

In this section, we introduce variable strength orthogonal arrays and then show that they are equivalent to partition-representations of a matroid and almost affine code. We then characterize matroidal entropy functions via variable strength orthogonal arrays.

### 3.1. Three Equivalent Definitions

#### 3.1.1. Variable-Strength Orthogonal Array

**Definition** **7.** *Given a loopless matroid M=(N,r) with r(N)≥2, a $v^{r(N)}\times n$ array T with columns indexed by N, entries from $Z_{v}$, is called a variable strength orthogonal array(VOA) induced by M with level v if for any A⊆N, $v^{r(N)}\times \left|A\right|$ subarray of T consisting of columns indexed by A satisfy the following condition: each row of this subarray occurs $v^{r(N)-r(A)}$ times. We also call such T a VOA(M,v).*

It can be seen that for each $v^{r(N)}\times \left|A\right|$ subarray $T^{\prime }$ of *T*, $v^{r(A)}$ distinct |A|-tuples in $Z_{v}^{\left|A\right|}$ occur in $T^{\prime }$. When *A* is independent, i.e., r(A)=|A|, they are exactly all tuples in $Z_{v}^{\left|A\right|}$.

**Example** **1.** *Let $M_{1}=\left(N,r_{1}\right)$ be a matroid with N={1,2,3,4,5} and rank function*

$$r_{1}\left(A\right)=\left\{\begin{array}{cc} \;|A|\; & |A|\leq 2\\ \;2\; & A\in \{\{1,2,3\},\{3,4,5\}\}\\ \;3\; & o.w. \end{array}\right.$$

*Then*

$$\begin{array}{ccccc} 0 & 0 & 0 & 0 & 0\\ 0 & 1 & 1 & 0 & 1\\ 1 & 0 & 1 & 0 & 1\\ 1 & 1 & 0 & 0 & 0\\ 0 & 0 & 0 & 1 & 1\\ 0 & 1 & 1 & 1 & 0\\ 1 & 0 & 1 & 1 & 0\\ 1 & 1 & 0 & 1 & 1 \end{array}$$

*is a $\mathrm{VOA}(M_{1},2)$.*

For a matroid *M*, let C be the set of all its circuits. From the definition, it can be seen that a VOA(M,v) is an $\mathrm{OA}(v^{r(N)};t,n,v)$ with $t=\min_{C\in C}\left|C\right|-1$, and so it has index $\lambda =v^{r(N)-t}$. For the matroid $M_{1}$ in Example 1, as r(N)=3 and smallest circuits {1,2,3} and {3,4,5} have size 3, the VOA(M,v) is an OA(8;2,5,2) and index λ=2.

However, on the other hand, two $\mathrm{OA}(\lambda v^{t};t,n,v)$s may be VOAs induced by two distinct matroids as long as they have the same rank and the same size of the smallest circuit. This is because the rank of a matroid provides richer parameters in VOA description than strength and index in OA description. The VOA description provides accurate information on the vary of strength on different set of columns of the array. This is why we term it “variable strength orthogonal array”.

**Example** **2.** *Let $M_{2}=\left(N,r_{2}\right)$ be a matroid with N={1,2,3,4,5} and rank function*

$$r_{2}\left(A\right)=\left\{\begin{array}{cc} \;2\; & A=\{1,2,3\}\\ \;|A|\; & |A|<3\\ \;3\; & \;o.w.\; \end{array}\right.$$

*Then $\mathrm{VOA}(M_{1},v)$ and $\mathrm{VOA}(M_{2},v)$ are both $\mathrm{OA}(v^{3};2,5,v)$.*

However, when the matroid is uniform, the two descriptions are equivalent. For a uniform matroid $U_{t,n}$, as any circuit has size t+1, a $\mathrm{VOA}(U_{t,n},v)$ has strength *t* and index λ=1, i.e., an OA(t,n,v). On the other hand, it can be seen any OA(t,n,v) is a $\mathrm{VOA}(U_{t,n},v)$. So in the following of this paper, we write $\mathrm{VOA}(U_{t,n},v)$ as OA(t,n,v) for simplicity.

#### 3.1.2. Partition-Representation of A Matroid

We will see that a partition-representation of a matroid *M* with degree *v* defined in [8] is equivalent to an VOA(M,v).

**Definition** **8.** *Let M=(N,r) be a matroid with ground set N and rank function r. Let $v\in Z^{+}$. The matroid M is partition representable of degree v if there exist a finite set *Ω* of cardinality $v^{r(N)}$ and partitions $\xi_{i}$ of *Ω*, i∈N, such that for any A⊆N, the meet-partition $\xi_{A}=\wedge_{i\in A}\xi_{i}$ has $v^{r(A)}$ blocks all the same cardinality.*

Let Ω be the set of all rows of an VOA(M,v). Let $\xi_{i}$ be a partition of Ω such that the rows in each block of $\xi_{i}$ have the same entry in the *i*-th column. It can be seem that $\xi_{i},i\in N$ is a partition-representation of *M* with degree *v*.

On the other hand, let $\xi_{i},i\in N$, be a partition-representation of a loopless matroid *M* with degree *v*, living on Ω. As each $\xi_{i}$ has *v* blocks, we label them from 0 to v−1. Now for each x∈Ω, it is labelled by an |N|-tuple $\left(x_{i},i\in N\right)$ where $x_{i}$ is the label of the block of $\xi_{i}$ to which x belong. Let *A* be an array whose rows are exactly the labels of all x∈Ω. It can be checked that *A* is an VOA(M,v).

#### 3.1.3. Almost Affine Codes

Almost affine codes were introduced in [9]. For vector space $F_{q}^{N}$ over finite field $F_{q}$, where *q* is a prime power, a linear subspace of $F_{q}^{N}$ forms a linear code of length *n*, while each coset of a linear code are called an affine code. For an affine code $C\subset F_{q}^{N}$ and any A⊆N, let $C_{A}$ be the projection of C onto $F_{q}^{A}$, it can be seen that $|C_{A}|$ is a power of *q*. But there are other codes satisfy this property even if they are not codes over a finite fields.

**Definition** **9.** *For a set of v symbols, say $Z_{v}$, $C\subseteq Z_{v}^{N}$ is called an* almost affine code *if*

(1) $$r\left(A\right):=\log_{v}\left|C_{A}\right|$$

*is an integer for all A⊆N.*

For any almost affine code C, (N,r) forms a matroid *M*, where the rank function r is defined in (1). We call such almost affine code an (M,v) (almost affine) code.

For an (M,v) code, if *M* is a uniform matroid $U_{t,n}$, it coincides with an (n,t,v) maximum distance separable (MDS) code.

By checking the definition of a VOA(M,v) and an (M,v) almost affine code, it can be seen that rows of a VOA(M,v) are exactly codewords of an (M,v) almost affine code and vice versa. In particular, the rows of a OA(t,n,v) are exactly codewords of an (n,t,v)-MDS code and vice versa.

If there exists an (M,v) almost affine code, *M* is called almost affinely representable with degree *v*.

### 3.2. Characterizing Matroidal Entropy Functions via VOA

Given a random vector $\left(X_{i},N\right)$, let $p_{X_{N}}\left(\cdot \right)$ denote its joint probability mass function, and for any A⊆N, $p_{X_{A}}\left(\cdot \right)$ be the marginal distribution function on *A*. Without loss of generality, we assume each random variable $X_{i}$ is distributed on $Z_{v_{i}}$ and for each $x\in Z_{v_{i}}$, $p_{X_{i}}\left(x\right)>0$.

**Theorem** **1.** *A random vector $X=(X_{i}:i\in N)$ characterizes the matroidal entropy function logv·M for a connected matroid M=(N,r) with rank r(N)≥2 if and only if the random variable X is uniformly distributed on the rows of a VOA(M,v).*

**Proof.**  Given a VOA(M,v), randomly pick a row from it according to the uniform distribution. Let $X_{i},i\in N$, be the random variable of *i*-th entries of picked *n*-tuple. For any A⊆N, consider the $v^{r(N)}\times \left|A\right|$ subarray of the VOA(M,v) consisting of columns indexed by *A*. By definition, it contains $v^{r(A)}$|A|-tuples in $Z_{v}^{\left|A\right|}$ as rows with each $v^{r(N)-r(A)}$ times. Hence $h_{X}\left(A\right)=\log v\cdot r\left(A\right)$. It proves that X characterizes logv·M and thus the “if part” of the theorem.

For the “only if part”, let $X=(X_{i}:i\in N)$ be a characterizing random vector of logv·M. Take C⊆N be a circuit of *M* of with cardinality $n^{\prime }\geq 3$. WLOG, we asume $C=\{1,2,\cdots ,n^{\prime }\}$. Then for each A⊊C, $X_{i},i\in A$ are mutually independent, and for each i∈C, $X_{i}$ is a function of $\left(X_{j}:j\in C-i\right)$. Then for each A⊊C and $x_{i}\in Z_{v_{i}},i\in A$, $p_{X_{A}}\left(x_{i}:i\in A\right)=\prod_{i\in A}p_{X_{i}}\left(x_{i}\right)$, and for each i∈C, $p_{X_{C}}\left(x_{j}:j\in C\right)=p_{X_{C-i}}\left(x_{j}:j\in C-i\right)$. In particular,

(2) $$p_{X_{C}}\left(x_{1},x_{2}\cdots ,x_{n^{\prime }}\right)=p_{X_{C-1}}\left(x_{2},\cdots ,x_{n^{\prime }}\right)=p_{X_{2}}\left(x_{2}\right)...p_{X_{n^{\prime }}}\left(x_{n^{\prime }}\right)$$

and

(3) $$p_{X_{C}}\left(x_{1},x_{2}\cdots ,x_{n^{\prime }}\right)=p_{X_{C-2}}\left(x_{1},x_{3}\cdots ,x_{n^{\prime }}\right)=p_{X_{1}}\left(x_{1}\right)p_{X_{3}}\left(x_{3}\right)...p_{X_{n^{\prime }}}\left(x_{n^{\prime }}\right)$$

Equating (2) and (3), we have

(4) $$p_{X_{1}}\left(x_{1}\right)=p_{X_{2}}\left(x_{2}\right).$$

Let $x_{1}^{\prime }\in X_{1}$ and $x_{1}^{\prime }\neq x_{1}$, with the same argument, we have

(5) $$p_{X_{1}}\left(x_{1}^{\prime }\right)=p_{X_{2}}\left(x_{2}\right).$$

As $x_{1}$ and $x_{1}^{\prime }$ are arbitrary chosen from $Z_{v_{1}}$, $X_{1}$ is uniformly distributed on it. Since $h_{x}\left(\left\{1\right\}\right)=\log v$, $v_{1}=v$. By symmetry, for all i∈C, $X_{i}$ is uniformly distributed on $Z_{v}$. Since *M* is a connected matroid with r(N)≥2, each element is contained in a circuit with size not less than 3. Hence for all i∈N, $X_{i}$ is uniformly distributed on $Z_{v}$. Thus, in the following X can be considered to distributed on $Z_{v}^{N}$ and for any A⊆N, $X_{A}$ is distributed on $Z_{v}^{A}$.

Now let B⊆N be a base of *M*, i.e., r(B)=|B|=r(N). Since $h_{X}\left(B\right)=\log v\cdot r\left(N\right)$, any |B|-tuple $x_{B}$ in $Z_{v}^{B}$, $p_{X_{B}}\left(x_{B}\right)=v^{-r(N)}>0$. It implies that there exists at least $v^{r(N)}$*n*-tuples $x\in Z_{v}^{N}$ with $p_{N}\left(x\right)>0$ and the marginal distribution of them on *B* is uniform. As $h_{X}\left(N\right)=h_{X}\left(B\right)=\log v\cdot r\left(N\right)$, each $x\in Z_{v}^{N}$ with $p_{N}\left(x\right)>0$ is uniquely determined by their entries indexed by *B*, and so there are exactly $v^{r(N)}$*n*-tuples $x\in Z_{v}^{N}$ with $p_{N}\left(x\right)=v^{-r(N)}$ and other *n*-tuples has zero probability. Furthermore, for any A⊆N, as $h_{X}\left(A\right)=\log v\cdot r\left(A\right)$, by taking sub-tuples indexed by *A* of these $v^{r(N)}$*n*-tuples, we obtain $v^{r(A)}$ distinct |A|-tuple in $Z_{v}^{\left|A\right|}$, each of which occur exactly $v^{r(N)-r(A)}$ times. Therefore, if we put these *n*-tuples in an array and each as a row, they form a VOA(M,v).  □

**Corollary** **1.** *A random vector $X=(X_{i}:i\in N)$ characterizes matroidal entropy function $\log v\cdot U_{t,n}$ with 2≤t≤n−1 if and only if random variable Y=X is uniformly distributed on the rows of an OA(t,n,v).*

## 4. P-Characteristic Set of Matroids with Order n≤5

Rank 1 matroids of order *n* are exactly those matroids containing $U_{1,n^{\prime }}$ on $N^{\prime }\subseteq N$ as a submatroid and other elements loops. Let *X* be an arbitrary random variable. Let $\left(X_{i}:i\in N\right)$ be defined by

$$X_{i}=\left\{\begin{array}{cc} X\; & i\in N^{\prime }\\ a\;\mathrm{constant}.\; & o.w. \end{array}\right.$$

It can be seen that $\left(X_{i}:i\in N\right)$ characterizes h on the ray $\left\{h\in H_{n}:h=c\cdot M\right\}$ as long as we let H(X)=c.

Armed with the results of orthogonal arrays in Section 2.2 and Theorem 1, we can characterize the matroidal entropy functions logv·M for a connected matroid *M* with rank ≥2. In this section, we determine the p-characteristic set $\chi_{M}$ for all connected matroids M=(N,r) with rank r(N)≥2 and order n≤5. For a disconnected matroid *M* with each connected component $M_{i}$ rank ≥2, $\chi_{M}$ is the intersections of all $\chi_{M_{i}}$. Thus, it is sufficient to consider connected matroids and take them as building blocks. It matches the fact that matroidal entropy functions indexed by a connected matroid live on an extreme rays of $\Gamma_{n}$ (see Lemma 1), while those indexed by a disconnected matroid can be written as the sum of the matroidal entropy functions indexed by its connected components.

Among all connected matroids, we only need to consider those simple matroids since the p-characteristic set of a matroid is the same as its simplification. For a matroid M=(N,r) and its simplification $M^{\prime }=\left(N^{\prime },r^{\prime }\right)$ with $N^{\prime }\subseteq N$, if $\left(Y_{j}:j\in N^{\prime }\right)$ characterizes $\log v\cdot M^{\prime }$, for each parallel class *A*, let $X_{i}=Y_{j}:i\in A$ where *j* is the only element in $A\cap N^{\prime }$, and let $X_{i}$ be a constant if *i* is a loop of *M*. Then $\left(X_{i}:i\in N\right)$ characterizes logv·M. On the other hand, if $\left(X_{i}:i\in N\right)$ characterizes logv·M, by the reverse method, we obtain $\left(Y_{j}:j\in N^{\prime }\right)$ characterizing $M^{\prime }$. Hence they have the same p-characteristic set.

Non-isomorphic simple matroids with order ≤8 is listed in [17] (A simple matroid is also called a combinatorial geometry.). Here we consider connected simple matroids with rank r(N)≥2 and order n≤5. Before that we first consider $U_{n-1,n}$ for general n≥3. By Lemma 2 and Theorem 1, we have the following proposition.

**Proposition** **1.** $$\chi_{U_{n-1,n}}=\left\{v\in Z:v\geq 2\right\}.$$

When n=3, the case $U_{2,3}$ is also proved by Zhang-Yeung [1] as we discussed in Section 1. As $U_{2,3}$ is the only case we need to consider for matroids with oder n=3, in the following we discuss the cases for n=4 and 5.

### 4.1. n=4

For n=4, besides $U_{3,4}$, one more matroid we need to consider is $U_{2,4}$. By Theorem 1 together and Lemma 3, we have the following propositions.

**Proposition** **2.** $$\chi_{U_{2,4}}=\left\{v\in Z:v\geq 3,v\neq 6\right\}.$$

### 4.2. n=5

For n=5, besides $U_{4,5}$, there are four more matroids we need to consider, namely, $U_{2,5}$, $U_{3,5}$, $M_{1}$ defined in Example 1 and $M_{2}$ defined in Example 2.

For $U_{2,5}$, by Theorem 1 and Lemma 4, we have the following propositions.

**Proposition** **3.** *For $U_{2,5}$, $2,3,6\notin \chi_{U_{2,5}}$ and $Z^{+}\setminus \left\{2,3,6,10\right\}\subseteq \chi_{U_{2,5}}$.*

For $U_{3,5}$, by Theorem 1 and Lemmas 5–7, we have the following propositions.

**Proposition** **4.** *For $U_{3,5}$, $2,3,6\notin \chi_{U_{3,5}}$ and $V\subseteq \chi_{U_{2,5}}$, where $V=V_{1}\cup V_{2}$ and*

*1.*  $V_{1}=\left\{v\geq 4:v≢2\;\left(\mathrm{mod}\;4\right)\right\}$
*2.*  *$V_{2}$ is the set of v≡2(mod4) such that*
  - *v=35xg+5, if x≡1(mod4);*
  - *v=35xg+7, if x≡3(mod4)*
*where x is an arbitrary odd positive integer, and g is an arbitrary positive integer whose prime power factors are all ≥7 such that g≡3(mod4).*

For $M_{1}$, we give a $\mathrm{VOA}(M_{1},2)$ in Example 1, thus $2\in \chi_{M_{1}}$. We will have in the following proposition on the existence of $\mathrm{VOA}(M_{1},v)$ for an arbitrary v≥2.

**Proposition** **5.** $$\chi_{M_{1}}=\left\{v\in Z:v\geq 2\right\}.$$

**Proof.**  For any v≥2, let $\left(y_{1},y_{2},y_{3}\right)$ be any 3-tuple in $Z_{v}^{3}$. Now given $\left(y_{1},y_{2},y_{3}\right)$, let $x_{1}=y_{1}$, $x_{2}=y_{2}$, $x_{3}=y_{1}+y_{2}$, $x_{4}=y_{3}$ and $x_{5}=x_{1}+x_{2}+x_{3}$, we obtain a 5-tuple $\left(x_{1},x_{2},x_{3},x_{4},x_{5}\right)$. Run out of all $\left(y_{1},y_{2},y_{3}\right)\in Z_{v}^{3}$, it can be checked that the resulting $v^{3}$ 5-tuples form a VOA(M,v). Since v≥2 is arbitrary, the proposition holds.  □

The following proposition determines the p-characteristic set of $M_{2}$.

**Proposition** **6.** $$\chi_{M_{2}}=\left\{v\in Z:v\geq 3,v\neq 6\right\}.$$

**Proof.**  We prove that if there exist an OA(2,4,v), then there exists a $\mathrm{VOA}(M_{2},v)$, and vice versa. It implies that $\chi_{M_{2}}=\chi_{U_{2,4}}$ and hence the proposition.

Now assume there is an OA(2,4,v) with columns $a_{i},i=1,2,3,4$. So each $a_{i}$ is a $v^{2}$-vector. Let $b_{i},i=1,2,3,4,5$ be $v^{3}$-vectors defined as follows.

$$b_{i}\left(kv^{2}+j\right)=\left\{\begin{array}{cc} \;a_{i}\left(j\right)\; & i=1,2,3\\ \;a_{4}\left(j\right)+k\;\mathrm{mod}\;v\; & i=4\\ \;k\; & i=5 \end{array}\right.$$

for each $j=1,2,\cdots ,v^{2}$ and k=0,1,⋯,v−1. It can be checked that $b_{i},i=1,2,3,4,5$ form a $\mathrm{VOA}(M_{2},v)$.

On the other hand, assume there is a $\mathrm{VOA}(M_{2},v)$ with columns $b_{i},i=1,2,3,4,5$. As r({5})=1 and r(N)=3. The fifth column of $\mathrm{VOA}(M_{2},v)$ contains each $i\in Z_{v}$$v^{2}$ times. Rearrange the rows of $\mathrm{VOA}(M_{2},v)$ such that the first $v^{2}$ entries of $b_{5}$ are zeros, i.e., $b_{5}\left(j\right)=0$ for $j=1,2,\cdots ,v^{2}$. Let $a_{i}=1,2,3,4$ be $v^{2}$-vectors and $a_{i}\left(j\right)=b_{i}\left(j\right)$ for $j=1,2,\cdots ,v^{2}$. Then it can be checked that $a_{i}=1,2,3,4$ form an OA(2,4,v).  □

## 5. Discussion

### 5.1. Applications

Matroidal entropy functions and its characterizations have many potential applications in information theory. In the following we discuss the applications to network coding and secret sharing.

#### 5.1.1. Network Coding

A method of building networks from matroids was given in [18]. In a matroidal network *G*, messages generated in the source nodes and transmitted on the edges are mapped to the ground set of a matroid *M* (See Section V.B of [18]). By the same mapping, a VOA(M,v) with v≥2 can be considered as a (1,1) coding solution with alphabet size *v* of the network *G*. This coding solution is scalar but may not need to be linear.

#### 5.1.2. Secret Sharing

Let *M* be a connected matroid with rank ≥2 and *N* be its ground set. Let 1∈N be the special element. Let $A_{m}=\left\{C\setminus \left\{1\right\}:1\in C,C\;\mathrm{is}\;a\;\mathrm{circuit}\;\mathrm{of}\;M\right\}$ and A={A⊆N:$\exists B\in A_{m}\;s.t.\;B\subseteq A\}$. It can the checked that a VOA(M,v) forms an ideal secret sharing scheme of the access structure A, where the dealer is indexed by 1 and other participants are indexed by x∈N∖{1}. Such constructions can be seen in literatures such as [19,20,21,22,23].

### 5.2. Further Research

In this paper, we review and developed correspondences among matroidal entropy functions, connected matroids with rank ≥2, variable strength orthogonal arrays and almost affine codes. These correspondences can make them benefit from each other, and therefore yield more research topics in the following facets.

1. Results of orthogonal arrays can be leveraged to characterize matroidal entropy functions as we do in Section 4 for those of order ≤5.
2. Abundant tools in matroid theory can be used to study matroidal entropy functions, VOAs and almost affine codes. For example, in the proof of Lemma 5 and Proposition 6, we implicitly use the fact that $U_{2,4}$ is minor of $U_{2,5}$ and $M_{2}$, and $U_{2,4}$ is a forbidden minor for characteristic 2 and 6.
3. Matroid representability is an important and well-studied area in matroid theory. See [11], (Chapter 6). A matroid M=(N,r) is called representable over a field F if there exists a matrix *T* with entries in F whose columns are indexed by *N*, and for each A⊆N, the rank of the submatrix consisting of the columns indexed by *A* is equal to r(A). As we discussed in Section 3.1.2 and Section 3.1.3, a matroid is called partition-representable [8] or almost affinely representable [9] with degree *v* if there exists a VOA(M,v). Obviously, an $F_{q}$-representable matroid is also partition-representable with degree *q*. However, the converse of the statement may not hold in general.
4. The construction of an OA(t,n,v) is also an important problem in combinatorial design. For some parameters, say OA(2,5,10), the problem is extremely difficult. The definition of VOA provides more tools to attack the problem.
5. Matroidal entropy functions induced by $U_{t,n}$ are called symmetric matroidal entropy functions. They are special cases of the *p*-symmetrical entropy functions, where *p* is the trivial partition of *N* with *N* being the only block. In general, for an arbitrary permutation group *G* on *N*, symmetries of an *G*-symmetric matroidal entropy function, i.e., an entropy function that is *G*-symmetric [24] and matroidal, can be utilized to construct its characterizing random vectors via VOA. [25].

## Data Availability

Not applicable.

## Abbreviations

The following abbreviations are used in this manuscript:

OA	Orthogonal array
VOA	variable strength orthogonal array
MDS	Maximum distance separable
WLOG	With loss of generality
